# Estrogen- and Progesterone (P4)-Mediated Epigenetic Modifications of Endometrial Stromal Cells (EnSCs) and/or Mesenchymal Stem/Stromal Cells (MSCs) in the Etiopathogenesis of Endometriosis

**DOI:** 10.1007/s12015-020-10115-5

**Published:** 2021-01-07

**Authors:** Dariusz Szukiewicz, Aleksandra Stangret, Carmen Ruiz-Ruiz, Enrique G. Olivares, Olga Soriţău, Sergiu Suşman, Grzegorz Szewczyk

**Affiliations:** 1 Department of General & Experimental Pathology with Centre for Preclinical Research and Technology (CEPT), Medical University of Warsaw, Pawinskiego 3C, 02-106 Warsaw, Poland; 2 Departamento de Bioquímica y Biología Molecular III e Inmunología, Facultad de Medicina, Universidad de Granada, Avenida de la Investigación, 11, 18016 Granada, Spain; 3 Laboratory of Radiotherapy, Tumor and Radiobiology, Prof. Dr. Ion Chiricuţă Oncology Institute, 34-36 Republicii St, 400015 Cluj-Napoca, Romania; 4 Department of Histology, Iuliu Hatieganu, University of Medicine and Pharmacy, Cluj-Napoca, Romania

**Keywords:** Endometrial stromal cells, Mesenchymal stem/stromal cells, Etiopathogenesis of endometriosis, Epigenetic modifications, Estrogen signaling, Estrogen receptors, Progesterone signaling, Progesterone receptors

## Abstract

Endometriosis is a common chronic inflammatory condition in which endometrial tissue appears outside the uterine cavity. Because ectopic endometriosis cells express both estrogen and progesterone (P4) receptors, they grow and undergo cyclic proliferation and breakdown similar to the endometrium. This debilitating gynecological disease affects up to 15% of reproductive aged women. Despite many years of research, the etiopathogenesis of endometrial lesions remains unclear. Retrograde transport of the viable menstrual endometrial cells with retained ability for attachment within the pelvic cavity, proliferation, differentiation and subsequent invasion into the surrounding tissue constitutes the rationale for widely accepted implantation theory. Accordingly, the most abundant cells in the endometrium are endometrial stromal cells (EnSCs). These cells constitute a particular population with clonogenic activity that resembles properties of mesenchymal stem/stromal cells (MSCs). Thus, a significant role of stem cell-based dysfunction in formation of the initial endometrial lesions is suspected. There is increasing evidence that the role of epigenetic mechanisms and processes in endometriosis have been underestimated. The importance of excess estrogen exposure and P4 resistance in epigenetic homeostasis failure in the endometrial/endometriotic tissue are crucial. Epigenetic alterations regarding transcription factors of estrogen and P4 signaling pathways in MSCs are robust in endometriotic tissue. Thus, perspectives for the future may include MSCs and EnSCs as the targets of epigenetic therapies in the prevention and treatment of endometriosis. Here, we reviewed the current known changes in the epigenetic background of EnSCs and MSCs due to estrogen/P4 imbalances in the context of etiopathogenesis of endometriosis.

**Figure:**
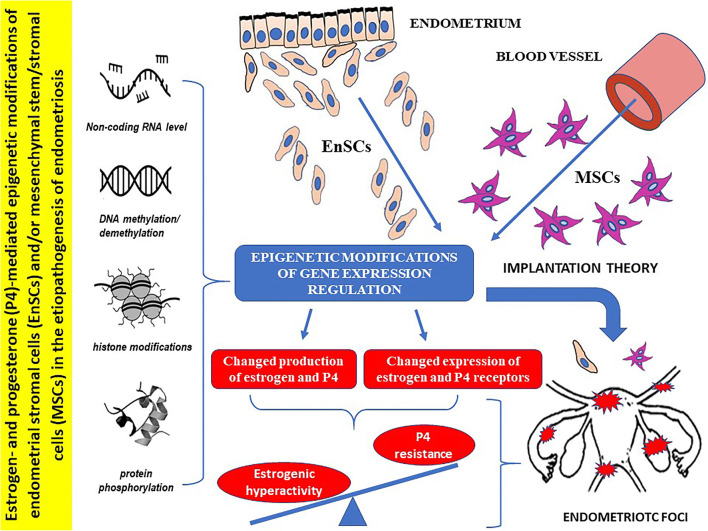
Graphical Abstract

Graphical Abstract

## Introductory Overview

### Endometriosis – Disorder Characteristics and Pathogenesis Theories

The term “endometriosis” refers to a condition in which endometrial tissue appears outside the uterine cavity [1]. Endometriosis can be either endopelvic or extrapelvic, depending on the location of endometrial tissue implantation. Abnormally located endometrial foci are primarily found in the pelvis, including ovaries, ovarian fossa, fallopian tubes, uterine wall (endometriosis interna or adenomyosis), broad ligaments, round ligaments, uterosacral ligaments, appendix, large bowel, ureters, bladder or rectovaginal septum [2, 3]. Extrapelvic locations of endometriosis are rare. However, several cases of endometriosis of upper abdomen, abdominal wall, abdominal scar tissue, diaphragm, pleura, pericardium, liver, pancreas, lower and upper respiratory tract tissue or even brain have been reported in the literature [3–6].

The tissue within the ectopic endometrium is biologically the same as basal intrauterine endometrial tissue, consisting of stroma cells, glands and smooth muscles [7]. The tissue is innervated and vascularized, including both blood and lymphatic networks [7, 8].

Because endometriosis cells express estrogen receptors (ERα, ERβ and GPER) and P4 receptors (PR-A and PR-B), they grow and undergo cyclic proliferation and breakdown similar to the endometrium [9, 10]. Local inflammatory reactions potentially caused by the bleedings may predispose to the occurrence of pain and more serious complications related to fibrosis, scar tissue formation and adhesions during repair processes [1, 11]. However, despite of the evidence for a relationship between endometriosis and inflammation, it is not clear whether the inflammatory process favors the development of endometriosis foci or the endometriosis foci induce the inflammatory process [12–14]. In the majority of endometriosis cases, pelvic pain, especially associated with menstruation, significantly compromises the quality of life of affected women [15]. Moreover, in addition to pain-related dysmenorrhea and dyspareunia, endometriosis reduces the ability to get pregnant and to have a successful pregnancy outcome [16]. It has also been observed that women with endometriosis have a higher incidence of cancer and autoimmune diseases [13, 17].

Endometriosis is a multifactorial disease with the involvement of genetic, immunological, hormonal, anatomical and environmental factors in different proportions [12–14]. The immune system is responsible for eliminating cells that are located in ectopic sites, and the failure of this elimination in endometriosis is due either to resistance of endometriotic cells to be eliminated by immune cells or to a deficit in the immune response [13, 18]. Endometriosis is known as an estrogen-dependent and P4-resistant process [19]. Numerous studies have shown that endometriosis is associated with aberrant growth and loss of sensitivity to apoptosis of endometrial tissue cells. Factors contributing to apoptosis resistance include increased expression of anti-apoptotic proteins, such as Bcl-2, c-IAP1, and c-IAP2, in ectopic endometrial cells compared to eutopic endometrial cells [20], which may explain their survival in ectopic foci and their resistance to elimination by apoptosis-inducing processes or by immune cells. The activating effect of estrogen on endometriotic cells may cause the anti-apoptotic status of these cells [21]. There are two types of endometriotic cells, namely epithelial and stromal, and the reported alterations tend to affect both cell types. It is not possible to affirm whether these alterations are intrinsic to the endometriotic cells or induced by their ectopic location [21].

Despite several decades of intensive investigation into the underlying etiology and pathogenesis of endometriosis, the current understanding of the disease remains unclear. Several theories for the pathogenesis of endometriosis have been elaborated or updated in recent years, including implantation and metaplasia of Müllerian-type epithelium (coelomic metaplasia) theories as well as the induction theory (a combination of the previous two theories) that assumes the influence of unidentified substances released from shed endometrium inducing formation of endometriotic tissue from undifferentiated mesenchyme [12, 22]. It has been recently proposed that endometriosis develops from stem cells derived from bone marrow, which would also explain extraperitoneal endometriosis lesions [23, 24].

**Fig. 1:**
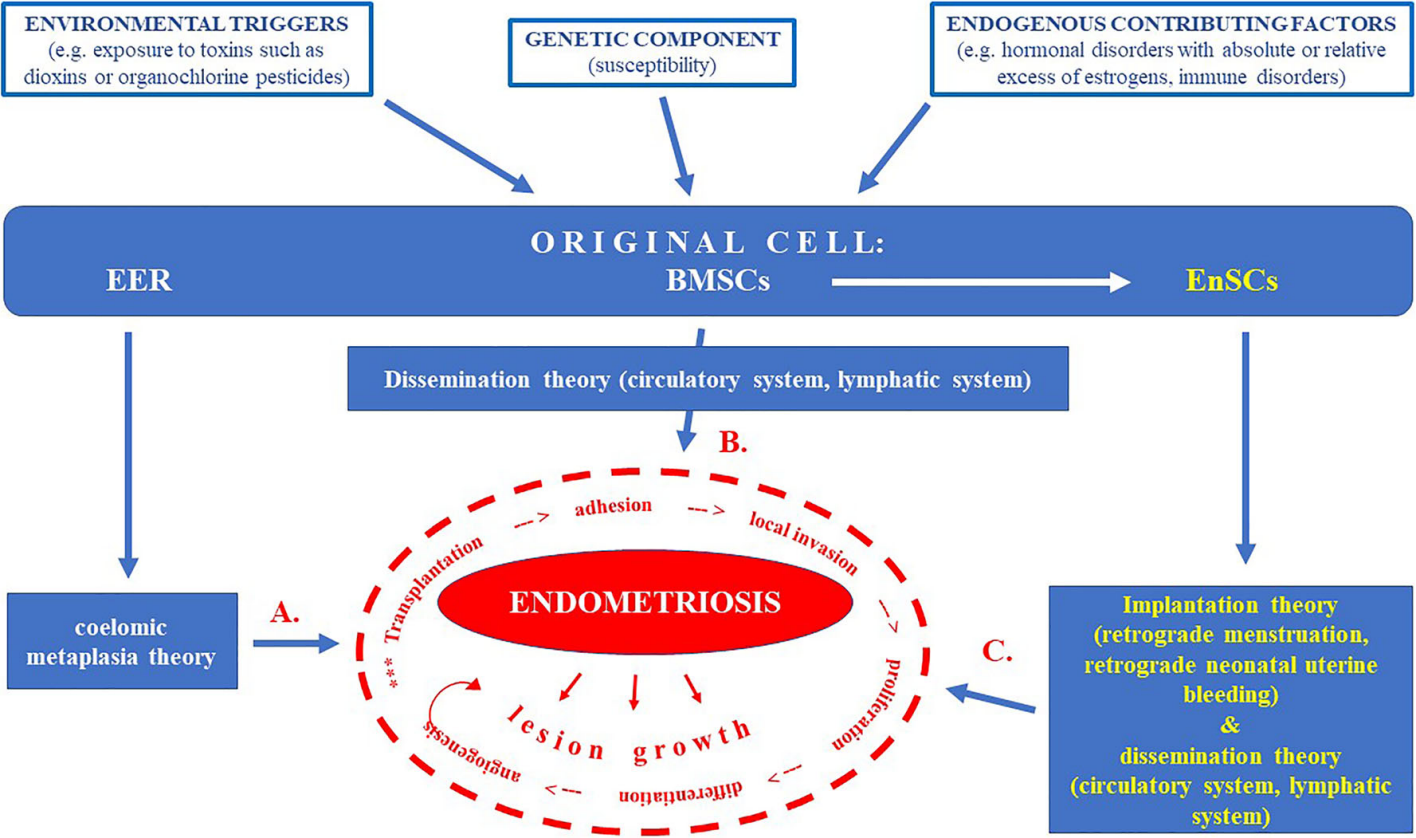
Theories on the pathogenesis of endometriosis related to stem cells. Endometrial stem cells related pathway marked in yellow. (EER – embryonic epithelial remnants; BMSCs – bone marrow stem/progenitor cells; EnSCs – endometrial stromal cells). **A.** Endometriosis may originate from the metaplasia of EER (e.g., from embryonic mullerian system) that are present in the mesothelial lining of the visceral and abdominal peritoneum; **B.** BMSCs could disseminate to ectopic sites via hematogenous and lymphatic spread (hematogenous or lymphatic metastases, respectively), accounting for the presence of endometriosis lesions in distant sites outside the pelvis, including the brain, lung, lymph nodes, extremities, spine and the abdominal wall; **C.** In retrograde (retroperitoneal) menstruation, menstrual blood containing EnSCs derived from BMSCs flows back through the fallopian tubes and into the pelvic cavity. This endometrial reflux is commonly observed during menstruation, but in certain conditions of defective cellular immunity EnSCs may implant and proliferate. In addition to implantation theory, hematopoietic and lymphatic dissemination of EnSCs is proposed

Retrograde transport of viable menstrual endometrial cells with retained ability to attach within the pelvic cavity (initially to the peritoneum), proliferate, differentiate and invade into surrounding tissue constitutes the rationale for the most widely accepted implantation theory. According to this theory, endometrial cells may also spread out through the lymphatic and/or the vascular system, resulting in formation of endometrial foci in more distant locations [18, 22]. In addition, evidence that the endometrium contains a particular population of cells with clonogenic activity that resembles properties of mesenchymal stem cells (MSCs), may shed new light on the implantation theory, suggesting a significant role of stem cell-based dysfunction in formation of the initial endometrial lesions [25, 26]. Some endometrial cell dysfunction may explain why the retrograde menstruation process frequently observed in healthy women is not associated with endometriosis initiation [27]. Theories on the pathogenesis of endometriosis related to stem cells are presented in Fig. 1.

Similar to the pathogenesis, considerable controversy remains regarding the prevalence, natural history and optimal treatment of endometriosis [16, 22, 28–30]. It is generally accepted that approximately 10% (range of 5 to 15%) of reproductive aged women suffer from endometriosis, whereas significantly higher percentages of endometriosis-related treatments (25 to 50%) have been administered amongst infertile female patients [28–30]. Moreover, the prevalence of endometriosis may be influenced by race/ethnicity [31].

### Endometrial Stem Cells

#### Endometrial/Decidual Stromal Cells

The most abundant cells in the human endometrium are endometrial stromal cells (EnSCs). During the secretory phase of the menstrual cycle, especially if pregnancy occurs, EnSCs or their equivalents in the decidua, i.e., decidual stromal cells (DSCs), are differentiated (decidualized) by the effect of P4 and other hormones. During this process, EnSCs or DSCs increase in size and change in shape from a fibroblastic appearance to a rounder morphology. Decidualized cells produce prolactin (PRL), insulin-like growth factor binding protein-1 (IGFBP-1) and other factors, such as IL15 [32]. Some authors consider EnSCs as precursor undifferentiated cells and DSCs as decidualized cells [33], thus creating confusion. Because the decidualization process occurs in both the decidua and the non-pregnant endometrium, there are precursor cells and decidualized cells in both tissues. The origin of EnSCs and DSCs as well as their cellular lineage ascriptions were unknown until recently. We isolated human EnSCs and DSCs, and we grew them in culture, establishing different cell lines that allowed us to identify the antigenic phenotype of these cells, define their functions and establish their origin and lineage (Table 1) [34, 37, 41, 51–53].

**Table 1:** Characteristics of decidual and endometrial stromal cells

ANTIGEN PHENOTYPE	References
CD45-, CD31-, CD3-, CD19- Endometrial stomal cell marker: CD10+ MSC/pericytes markers: CD13+, CD44+, CD90+, CD140b+, CD146+, α-SM actin+, nestin+, STRO-1+ eMSC markers: CD140b+, CD146+, SUSD2+	*Ruiz Magana*, 2020 [34]
DECIDUALIZATION	
Change from a fibroblastic to a rounder cell shape Change from a perivascular location to a location away from the blood vessels Secretion of PRL, IGFBP-1 and IL-15	*Ruiz Magana*, 2020 [34]; *Richards,* 1995 [32]
MSC CHARACTERISTICS	
MSC markers Mesenchymal differentiation Stem cell markers Clonogenicity	*Dimitrov*, 2010 [35]; *Dimitrov*, 2008 [36]; *Munoz-Fernandez*, 2018 [37]; *Muñoz-Fernández*, 2019 [38]; *Ruiz Magana*, 2020 [34]; *Shokri*, 2019 [39]
Hematopoietic cell supportive activity	*Alcayaga-Miranda*, 2015 [40]; *Blanco,* 2009 [41]
Inhibition of NK cell cytotoxicity	*Croxatto*, 2014 [42]; *Shokri*, 2019 [39]
Survival in xenotransplants	*Muñoz-Fernández*, 2019 [38];*Ye*, 2018 [43]
Therapeutic effects on immune-based diseases	*Muñoz-Fernández*, 2019 [38];*Xu*, 2018 [44]
PERICYTE CHARACTERISTICS	
Perivascular location of preDSCs and preEnScs	*Ferenczy*, 1983 [45]; *Munoz-Fernandez*, 2018 [37]; *Wynn*, 1974 [46]
Pericyte markers	*Munoz-Fernandez*, 2018 [37]; *Ruiz Magana*, 2020 [34]
Expression of angiogenic factors	*Alcayaga-Miranda*, 2015 [40]; *Munoz-Fernandez,* 2018 [37]
Cell contractility	*Kim,* 2020 [47]; *Munoz-Fernandez,* 2018 [37]
Chemotactic activity	*Hirota,* 2006 [48]; *Munoz-Fernandez,* 2018 [37]
Phagocytosis activity	*Cornillie,* 1985 [49]; *Ruiz,* 1997 [50]

Several groups have demonstrated in humans and mice that EnSCs and DSCs show immunological activities [34, 41, 53–55], suggesting that these cells may have a relevant role in the immunological interrelationship between the mother and fetus as well as in maternal–fetal tolerance (Table 1). In addition to the finding that EnSCs and DSCs are the same cell in two different physiological situations (non-gestation and gestation, respectively), it has been observed that DSCs are more responsive to decidualization, suggesting that the pregnancy environment enhances the capacity of stromal cells to decidualize [34]. Interestingly, this progression is blocked in endometriotic cells [23]. Stromal cells in endometriosis foci (eEnSCs) show an antigen phenotype equivalent to that of EnSCs and DSCs, but they do not fully decidualize [21]. During decidualization, EnSCs and DSCs undergo apoptosis [56], but eEnSCs are resistant to cell death [20].

#### Endometrial/Decidual Stromal Cells and Mesenchymal Stem/Stromal Cells

Based on the expression of STRO-1, a MSC marker, by DSCs, one of us (EGO) was the first to propose the relationship between DSCs and mesenchymal stem/stromal cells (MSCs) [52]. This possibility was subsequently confirmed by other groups for DSCs and EnSCs [35, 36]. In our experience, the phenotype and functionality of EnSC and DSC lines are identical. It has been observed that these cells express MSC-associated antigens and stem cell markers (OCT-4, NANOG and ABCG2), and under appropriate culture conditions, they have the ability to differentiate into osteoblasts, chondrocytes and adipocytes, indicating that EnSCs and DSCs are closely related to or derived from MSCs [35, 36, 38]. In the case of EnSCs, this possibility has been confirmed in women who have received a bone marrow transplant because donor cells (both stromal and epithelial cells) have been detected in their endometrium [57]. Precursors of DSCs and EnSCs (preDSCs and preEnScs) also correspond to MSCs in the human endometrium (endometrial MSCs, eMSCs) as reported by other authors, i.e., clonogenic, self-renewing, multipotent cells that can differentiate into adipogenic, osteogenic, chondrogenic and myogenic lineages. Similar to preDSCs and preEnSCs, eMSCs are CD146+, CD140b+ and SUSD2+, and they decidualize, are found in perivascular sites and have also been associated with pericytes [34, 37, 58]. MSCs may migrate from bone marrow to different tissues to give rise to different mesenchymal lineages as follows: fibroblasts, adipocytes, osteoblasts and myofibroblasts as well as EnSCs and DSCs in the uterus. Logically, cells derived from this same precursor share a number of common morphological, antigenic and functional characteristics as we have observed for DSCs and EnSCs [34, 37, 52, 59].

#### Endometriosis and Mesenchymal Stem/Stromal Cells

The relationship between EnSCs and MSCs may explain the appearance of endometriosis foci in distant sites, such as the skin, lung and brain, as well as cases of endometriosis in men, which are not attributable to retrograde menstruation but to “erroneous homing” of MSC-related precursors, which are transported by the blood from the bone marrow to extraperitoneal tissues. In addition, MSC-related precursors present in menstrual blood may reach the peritoneum by retrograde menstruation [23, 35]. Nevertheless, endometriosis foci (both peritoneal and extraperitoneal) contain both EnSCs and epithelial cells. These two cell types may reflect either the existence of a common precursor that gives rise to both epithelial and stromal cell lineages or to the existence of two independent precursors that develop in the bone marrow and then colonize the endometrium. Given these two possibilities, it is unlikely that two independent precursors from the bone marrow can each erroneously colonize an extraperitoneal tissue to produce extraperitoneal endometriosis foci. Thus, it is more likely that there is a single precursor that gives rise to both epithelial and stromal cells. The fact that both MSCs and EnSCs can differentiate into epithelial cells [58] supports the existence of a single precursor related to MSCs and EnSCs. This precursor may colonize the endometrium under normal conditions where it differentiates into epithelial and stromal cells. In endometriosis, the precursor may form both peritoneal and extraperitoneal foci, which differentiate into both epithelial and stromal cells. Similar to EnSCs and DSCs, bone marrow MSCs change from a fibroblastic to a rounder morphology and express PRL mRNA in the presence of P4 and cAMP (decidualization factors) in culture, but unlike EnSCs and DSCs, MSCs are unable to secrete PRL [53]. A similar process may occur in the case of eEnSCs, which undergo morphological modifications and express PRL mRNA when decidualized but are unable to secrete PRL [21]. These findings suggest that eEnSCs are closer to MSCs than to EnSCs and DSCs, and they also suggest that eEnSCs have lost, through a primary or induced mechanism, the ability to progress toward decidualized EnSCs [23].

#### Ectopic Tissues with Stromal and Immune Cells: Endometriosis

We have observed phenotypic and functional relationships between DSCs/EnSCs and stromal cells (SCs) in secondary lymphoid organs (SLOs). These SCs also derive from MSCs and contribute to lymphoid tissue organization by interacting with immune cells in SLOs, attracting these cells by secreting chemokines and inhibiting their apoptosis by producing anti-apoptotic factors [60]. In addition to their similarity in antigen phenotype with SCs, EnSCs and DSCs have chemotactic and anti-apoptotic properties similar to those of SCs [53, 54, 59, 61]. Although the decidua and endometrium cannot be considered SLOs (because they lack the characteristic compartmentalization in T and B zones), they may be considered non-lymphoid immune tissues equivalent to the skin. As shown for SCs, EnSCs and DSCs may also participate in the organization of the endometrium and decidua by attracting and interacting with leukocytes. Another aspect shared by EnSCs and SCs of SLOs is that both cell types have been detected in ectopic locations associated with inflammatory processes. Patients with rheumatoid arthritis frequently have lymphoid tissue in the synovium (a tertiary lymphoid organ) along with the presence of SCs [62]. Endometriosis may represent an equivalent ectopic situation for EnSCs despite the finding that endometriomas contain eEnSCs and a significant proportion of leukocytes, mainly macrophages [12, 13]. Similar to SCs, eEnSCs may attract leukocytes in ectopic areas, thereby contributing to the development of endometriomas.

#### Endometriosis as a Macrophage Disease

In a review of the involvement of macrophages in the pathogenesis of endometriosis [62], the authors argue that macrophage recruitment into lesions is not only an early event in the development of foci but is also a necessary step for the establishment of endometriotic lesions. Macrophages produce cytokines, growth factors and angiogenic factors that affect eEnSCs and contribute to the development of endometriomas. In other words, endometriosis arises from the crosstalk between eEnSC and macrophages. Although M2 macrophages have been observed in the peritoneum and in endometriosis foci [63], there is no consensus regarding the type of macrophage involved in the process. One possibility is that M1 macrophages contribute to the inflammatory environment, while M2 cells favor the angiogenesis that characterizes the disease [64]. eEnSCs may secrete chemokines that attract macrophages to the endometriosis foci. However, one of the most important contributors in intercellular communication are extracellular vesicles (EV) that transport molecules from one cell or tissue to another. EVs perform their function by interacting directly with receptors on the cell surface or by delivering their contents to the target cell by endocytosis, phagocytosis or membrane fusion. EVs contain a wide variety of cytoskeletal, cytosolic, plasma membrane and heat shock proteins. The presence of cytokines, miRNA and other ncRNAs have also been reported. Recent work has documented differences in the miRNA profile between exosomes released by EnSCs from patients with endometriosis and exosomes from normal endometrium [65]. More recently, work in a murine model of endometriosis has demonstrated the ability of exosomes produced by eutopic endometrial cells to regulate macrophage activity, favoring differentiation to M2 cells and reducing their phagocytic capacity [66].

Current therapeutic strategies for endometriosis are based primarily on hormone therapy. This treatment generates a hypoestrogenic state that leads to numerous side effects similar to those that occur during menopause, and it aggravates existing infertility problems in these women. Moreover, the success of this therapy depends on the location and type of endometriotic lesion. In the search for new approaches for treatment, a potentially informative avenue of study is to elucidate the molecular dialogue between eEnSCs and macrophages during the development of endometriosis as well as to identify the molecules that participate in this dialog to investigate the possibility of blocking them with antibodies or chemokine receptor antagonists.

### Epigenetic Modifications of Stem Cells

The unique nature of stem cells consists of three general properties as follows: capability of dividing and renewing themselves for long periods; unspecialization; and ability to differentiate into specialized cell types [67]. To that end, metabolism of stem cells and control of gene expression must be precise with rapid adjustment to changing conditions (e.g., hormonal status and menstrual cycle phase), including environmental factors [68, 69].

**Fig. 2:**
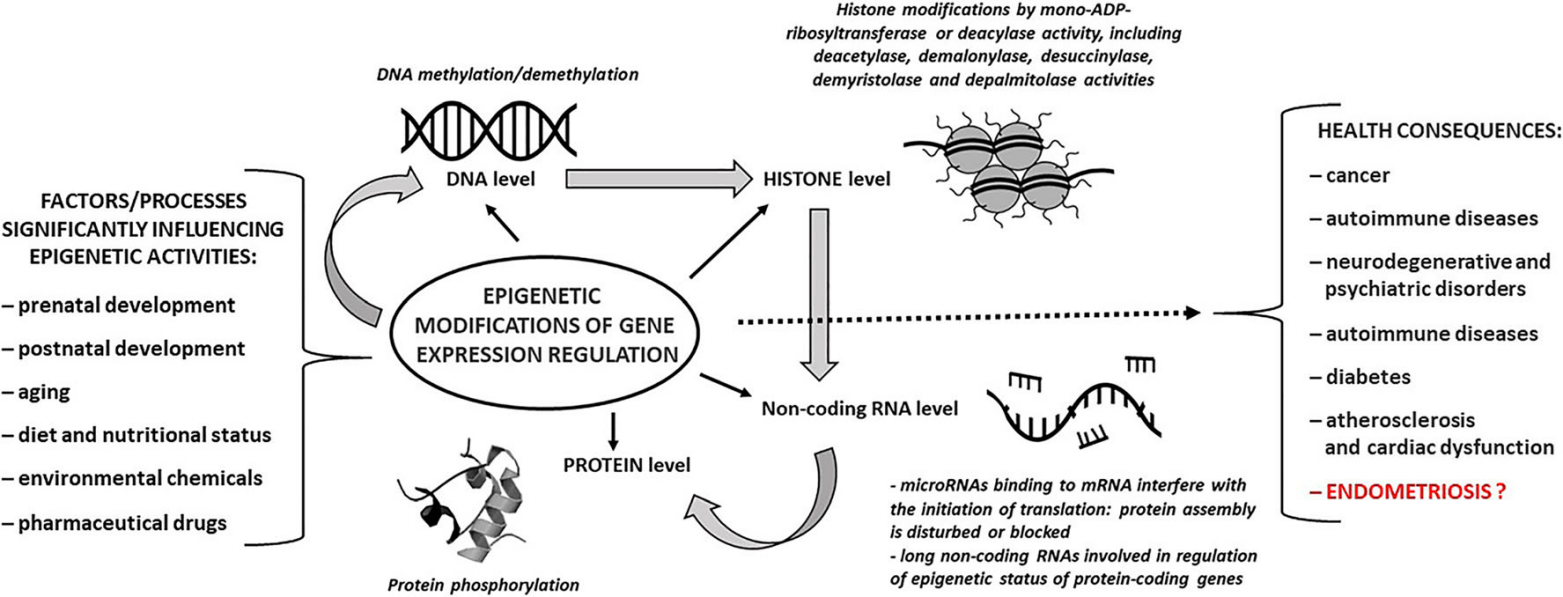
Main epigenetic mechanisms – an overview. Important factors influencing epigenetic activities and possible health consequences are also depicted

The control of gene expression has attracted the attention of researchers due to the possible induction of molecular mechanisms, resulting in epigenetic DNA modifications that involve changes in gene activity but not in DNA sequence [69, 70]. Thus, an epigenome consists of all chemical modifications to the DNA (e.g., methylation) and histone proteins (e.g., acetylation and succinylation) that regulate the expression of genes within the genome through chromatin condensation but without changes in the DNA nucleotide sequence [71]. Gene expression can be controlled through the action of repressor proteins that attach to silencer regions of the DNA, resulting in binding to mRNA and prevention of ribosome assembly. Small non-coding RNA (micro)molecules (miRNAs) containing approximately 20–22 nucleotides are abundant in many mammalian cell types and silence mRNAs by interfering with their translation. miRNA-dependent RNA silencing and post-translational regulation of gene expression occur through one or more of the following processes: cleavage of the mRNA strand into two pieces; destabilization of mRNA through shortening of its poly(A) tail; and less efficient translation of mRNA into proteins by ribosomes [72, 73]. Epigenetic regulation by miRNA targets approximately 60% of human genes [74]. The main epigenetic mechanisms and the most significant epigenetic factors are presented in Fig. 2.

Even without altered DNA sequence that lasts for multiple generations or only for the duration of the cell’s life, non-genetic factors may cause the organism’s genes to behave differently [75, 76]. Epigenetic change is a regular and natural occurrence in response to aging, the environment/lifestyle and disease state. This phenomenon is aimed to maintain genomic integrity [75, 77]. Accordingly, epigenetic homeostasis failure in the endometrial tissue may reflect local intrauterine abnormalities or generalized systemic pathology during repeated menstrual cycles or pregnancies due to endogenous causes (e.g., hormonal disorders) and/or exposure to some environmental risk factors [24, 78].

### Aim of Review

There has been increasing evidence in recent years that the role of epigenetic mechanisms and processes in the pathogenesis of various disease conditions in humans have been underestimated, including the unclear etiology of endometriosis [79–81].

In parallel with the progress in the understanding of modification of gene expression without changing DNA sequence, abnormal differentiation of stem cells and their clonogenic and/or proliferative activities have attracted the attention of independent scientific teams as a significant cause of morbidity and mortality [82]. It has been proposed that hormone-mediated epigenetic modifications of the genome in EnSCs or even MSCs play an important role in etiopathogenesis of endometriosis [83]. The roles of excess estrogen and P4 resistance are crucial [19].

Thus, the aim of this review was to combine the current knowledge of the epigenetic background of EnSCs and MSCs and the changed properties due to estrogen/P4 imbalances in the context of etiopathogenesis of endometriosis.

## Main Female Sex Hormones and Epigenetic Modifications of EnSCs and/or MSCs in Endometriosis

Hormone release dynamics govern periodic growth and regression of the endometrium, creating an extraordinary model for controlled tissue remodeling. Following the implantation theory of endometriosis that assumes the possibility of EnSCs spreading out with the menstrual blood, the interplay between sex steroid hormones throughout the menstrual cycle and the expression of their receptors deserves attention. Moreover, the nature of endometriosis is estrogen-dependent and P4-resistant [83, 84]. Thus, significant changes in the functional characteristic of EnSCs may result from epigenetic aberration of the expression of respective genes, especially genes linked to estrogens and P4 activities [85, 86].

### Estrogen Production and Metabolism

Both eutopic endometrium and ectopic endometrial foci are the main target tissues for estrogens, the primary female sex hormones [84]. At this point, it is worth noting that endometrial or endometriotic intratissue estrogen concentrations do not reflect the corresponding serum levels. Absolute or relative excess of estrogens has been reported in endometriosis, especially local within the lesions [87]. Estrogen-dependent endometriosis is rarely diagnosed after menopause when the symptoms and endometriotic lesions are typically relieved [88]. Analogical reduction of the estrogen effect during pregnancy (overbalanced by P4) or pharmacological suppression of endogenous estrogen synthesis (e.g., by use of ethinylestradiol-containing combined oral contraceptive pills) are likely to diminish intensity of the disease [89, 90].

The member of the cytochrome P450 family (CYP) and the product of the CYP19A1 gene, aromatase (EC 1.14.14.1), also known as estrogen synthetase or estrogen synthase, is a unique rate-limiting enzyme in the biosynthesis of estrogens from androgen precursors. The androgenic substrates for aromatase, androstenedione, testosterone and 16-hydroxytestosterone are converted into the following respective estrogens: estrone (E_1_), estradiol (E_2_; the most potent) and estriol (E_3_) [91]. Estradiol is an extremely strong mitogen for endometriotic tissue. Therefore, it is reasonable to assume that any alterations in aromatase activity will produce a shift in the balance between estrogenic and androgenic effects within the responsive tissues. Interestingly, it has been reported that growth of ectopic endometrial tissue requires high aromatase activity induction, which is normally not detectable in eutopic endometrium [92]. In contrast to endometriosis tissue, estrogens are not locally produced in endometrium. EnSCs produce estrogens, and the presence of P450 aromatase mRNA has been observed in EnSCs obtained from women with pelvic endometriosis [93]. Similar to breast cancer, aberrantly expressed aromatase in endometriotic stromal cells is stimulated by one of the best-known mediators of inflammation and pain, prostaglandin E_2_ (PGE_2_), via the promoter II region of the aromatase gene, resulting in local production of estrogen. Because estrogen itself upregulates cyclooxygenase 2 (COX-2) and therefore stimulates PGE_2_ formation, a positive feedback cycle is established [91–93].

There is evidence that a hyperestrogenic microenvironment within endometriotic lesions is a consequence of an epigenetic regulatory mechanism involving the aromatase gene located on chromosome 15q21. Multiple exons of this gene (CYP19A1) are potentially compatible with unique promotors that are present within the surroundings [94]. Alternative use of these exons ensures a precisely adjusted level of aromatase expression in the respective tissues. Endometriotic cells corresponding to EnSCs exploit identical aromatase promoters (promoters II, I.3 and I.6) as aromatase-free eutopic endometrial cells [95]. Considering that the endometriotic stromal cells share the same promoters with eutopic endometrial stromal cells, different expression of the aromatase gene indicates that an epigenetic regulatory mechanism inhibits this enzyme gene expression in healthy endometrium, whereas this effect is not present in endometriosis. CpG islands, the regions of the genome rich in promoters, are hypomethylated in endometriotic cells and hypermethylated in endometrial cells [96]. In particular, the differential expression of aromatase between eutopic normal endometrium and endometriotic foci may be due to the absence or presence, respectively, of the transcription factor, steroidogenic factor 1 (SF-1). In fact, methylation of CpG islands in the SF-1 gene, which spans from exon II to intron III, positively regulates its expression in stromal cells present in endometriosis, whereas hypomethylation of SF-1 gene CpG islands in normal endometrium is associated with drastically lower SF-1 levels [97, 98].

**Fig. 3:**
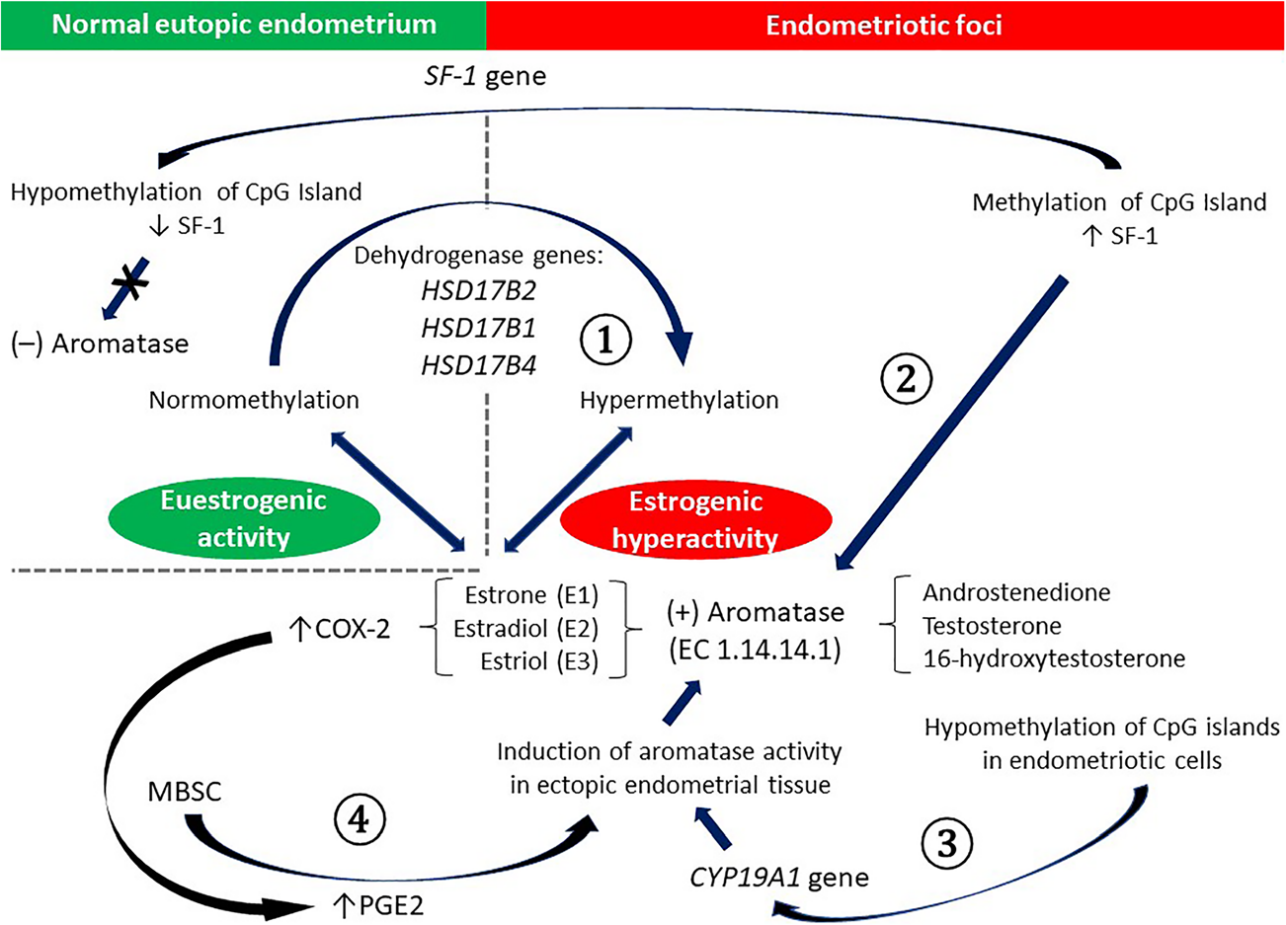
Interactions between estrogens and epigenetic modulators of estrogen signaling in endometriosis (see main text for details). ① - deficient 17β-hydroxysteroid dehydrogenases expression due to hypermethylation of the respective genes; ② - estrogenic hyperactivity caused by methylation of CpG island in the SF-1 gene; ③ - aromatase gene activation due to CpG islands hypomethylation; ④ - positive feedback: Estrogens → COX-2 → PGE2 → aromatase activity. *COX-2 – cyclooxygenase 2; CYP19A1 gene – gene coding aromatase (EC 1.14.14.1); MBSC – menstrual blood stem cells; PGE2 – prostaglandin E2; SF-1 – transcription factor steroidogenic factor 1*

Another abnormality pertaining to the estrogenic hyperactivity reported in endometriosis is caused by deficient 17β-hydroxysteroid dehydrogenase type 2 (17β-HSD2) expression. Physiologically, the conversion of adequate levels of 17β-estradiol to much less potent estrone is required to prevent accumulation of increasing quantities of estradiol in target tissues, including endometroid foci [99]. Such inactivation of 17β-estradiol is also regulated by DNA methylation, and it has been demonstrated that hypermethylation of the 17β-HSD2 gene body in ectopic stromal cells blocks the enzyme activity [100]. As mentioned above (see Chapter 1.3), DNA methylation is strictly linked to histone modifications and recruitment of histone deacetylases (HDACs) followed by chromatin condensation. The same epigenetic process (e.g., DNA methylation) is likely to influence activity of 17β-hydroxysteroid dehydrogenases type 1 and 4 (17β-HSD1 and 17β-HSD4, respectively), which are enzymes present in the human endometrium and EnSCs [101, 102]. The above interactions between estrogens and epigenetic modulators of estrogen signaling at the level of endometrial foci versus normal eutopic endometrium are shown in Fig. 3.

#### Estrogen Receptors (ERs) and Estrogen-Mediated Control of Epigenetic Mechanisms

Endometrial cells corresponding to MBSCs/MSCs and displaying stem cell markers, such as Oct-4, SSEA-4, Nanog and c-kit (CD117), simultaneously show expression of both main estrogen receptor isoforms (ERα and ERβ) and G protein-coupled estrogen receptor 1 (GPER), a member of the G protein-coupled receptor (GPCR) family [9, 10, 84, 103]. These ER subtypes are encoded by separate genes. Estrogen signaling is selectively regulated by the relative balance between ERα and ERβ expression in target organs. Although both ERα and ERβ are present in the endometrium, ERα is the primary mediator of the estrogenic action in this tissue [104]. Encoded by the *GPER* gene, the GPER protein is a member of the rhodopsin-like family of G protein-coupled receptors and is a multi-pass membrane protein that localizes (unlike the other members of the GPCR family) predominantly to the endoplasmic reticulum. GPER binds E_2_, resulting in mobilization of intracellular calcium and synthesis of phosphatidylinositol (3,4,5)-trisphosphate (PIP3) in the nucleus. Therefore, GPER is responsible for some of the rapid nongenomic effects that E_2_ exerts on cells [105]. It has been reported that GPER is significantly upregulated in endometriosis and during carcinogenesis, whereas epigenetic downregulation of GPER functions as a tumor suppressor in colorectal cancer [10, 106, 107].

There is no reason to assume that epigenetic regulation of estrogen receptors in EnSCs significantly differs from that observed in other estrogen-reactive tissues [98, 108, 109]. For instance, independent researchers have reported markedly higher levels of ERβ and lowered levels of ERα in human endometriotic stromal cells corresponding to EnSCs compared with EnSCs within eutopic endometrial tissues [110, 111]. These disorders have been linked to abnormally lowered methylation of a CpG island in the promoter region of the ERβ gene (*ESR2*) in endometriosis, resulting in ERβ overexpression. Bisulfite sequencing of this region has shown significantly higher methylation in primary endometrial cells versus endometriotic cells [112]. Moreover, treatment with a demethylating agent significantly increases ERβ mRNA levels in endometrial cells. High levels of ERβ, in turn, suppress ERα expression and response to E_2_ in endometriotic stromal cells via binding to non-classical DNA motifs in alternatively used ERα promoters [9]. Both in vitro and in vivo studies have confirmed induction of ERα expression in response to E_2_ in human endometrial stromal cells. However, in endometriotic foci, abnormally high quantities of E_2_ resulting from the local aromatase overactivity in addition to epigenetic upregulation of ERβ in stromal cells may suppress the normal response pertaining to ERα expression [113]. Lowered expression of ERα observed in endometriosis may predispose to insufficient responsiveness to E_2_ with respect to progesterone receptor (PR) expression, thus contributing to secondary PR deficiency and P4 resistance, which is typically observed in women with this disorder [9, 85]. Considering that ERβ also regulates cell cycle progression, another contributing factor to proliferation of endometriotic foci should be expected [114]. Thus, alteration in DNA methylation may be included in the pathomechanism responsible for severely increased ERβ mRNA levels in EnSC- and/or MSC-derived endometriotic cells.

Another epigenetic mechanism that may explain extraordinarily higher ERβ and significantly lower ERα and PR levels in endometriotic stromal cells compared with endometrial stromal cells is connected to miRNAs. The Human Genome Project has demonstrated that approximately 80% of our DNA is transcribed in RNA molecules but that only 2% of the genome is translated into proteins [115]. The majority of the remaining RNA does not code for proteins but is processed to produce functional RNAs. One of the most intensively studied groups of non-coding RNAs is miRNAs. miRNAs are crucial regulators of gene expression in E_2_-treated human endothelial cells [116]. Similarly, studies using animal models and humans have confirmed the significant role played by miRNAs in endometrial physiology and pathology by modulating the levels of estrogen receptor expression during the different phases of the menstrual (endometrial) cycle [115, 117]. It has been reported that numerous miRNAs directly target ERα, but less information is available for miRNAs modulating ERβ and GPER [118–121]. However, it has been recently demonstrated that GPER-mediated downregulation of miR-148a expression through the GPER/miR-148a/HLA-G signaling pathway may mediate the development of ovarian endometriosis [122].

Interestingly, the effect of direct regulation of ER expression by miRNAs is to some extent balanced by the following coexisting opposite mechanism: ER-mediated regulation of miRNA expression. A recent study has shown that E_2_-treated human umbilical vein endothelial cells (HUVECs) have differentially regulated specific miRNAs via pathways related to both classical ERs (ERα and ERβ) and membrane-bound ERs (GPER) [116]. Among the most modified miRNA, miR-30b-5p, miR-487a-5p, miR-4710 and miR-501-3p were over-expressed after E2 treatment, while miR-378 h and miR-1244 were down-regulated [116]. Analysis of the identified miRNAs indicates that these two mechanisms (regulation of ER expression by miRNAs vs. regulation of miRNAs by ERs) act in a parallel manner.

**Fig. 4:**
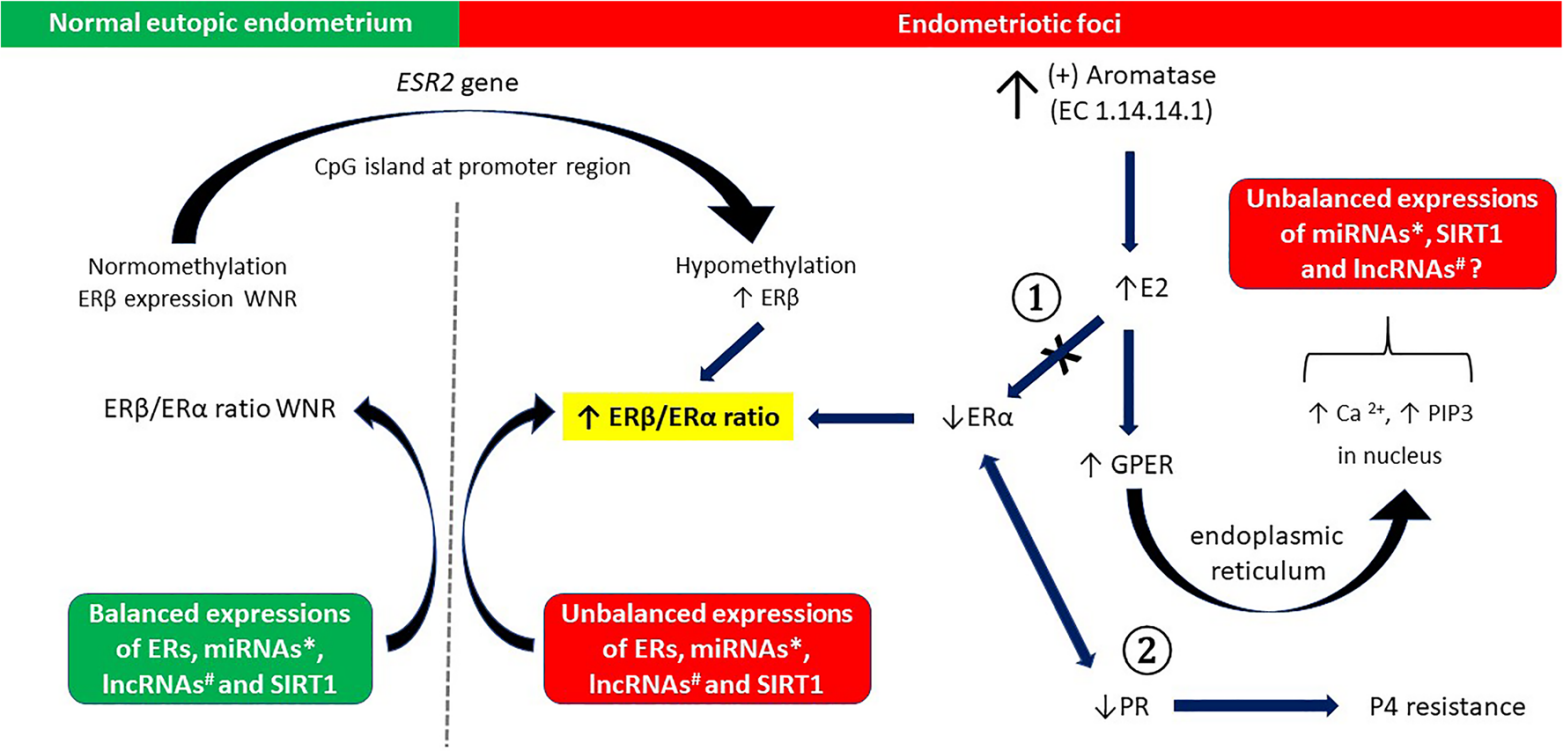
Normal vs. deranged estrogen receptors expression due to influence of epigenetic mechanisms: normal eutopic endometrium vs. endometriotic foci (see main text for details). Pathomechanism of P4 resistance is also depicted. ① - suppression of ERα expression in response to E_2_ via binding to non-classical DNA motifs in alternatively used ERα promoters; ② - decreased ERα expression-caused secondary PR deficiency leads to P4 resistance. *ERα, ERβ – estrogen receptor α and β, respectively; ESR2 gene – ERα gene; GPER – G protein-coupled estrogen receptor 1; PR – progesterone receptor; SIRT1 – sirtuin 1.* * Experimentally validated miRNAs that directly regulate ER gene expression microRNAs include miR-148a, miR-18a, miR-18b, miR-19a, miR-19b, miR-20b, miR-22, miR-130a, miR-193b, miR-206, miR-221, miR-222, miR-302c, let-7a, let-7b, let-7i, miR-92 [108, 122]; ER-mediated regulation of miRNA expression includes miR-30b-5p, miR-487a-5p, miR-4710, miR-501-3p, miR-378 h, miR-1244 [116]. ^#^ List of dysregulated ERs-associated lncRNAs detected in humans includes TMPO-AS1, LINC01116, H19, LASER1, MIR2052HG, LINC00707, LncRNA-Glu, LINC00472, LncRNA-RoR, NEAT1, MTA1, LncSHGL, HOTAIR [126]

In addition to miRNAs, some transcripts longer than 200 nucleotides lacking protein coding potential and transcribed by the RNA polymerase II (RNA Pol II), which are known as long non-coding RNAs (lncRNAs), may affect estrogen signaling by regulating the epigenetic status of protein-coding genes [123]. Together with the research progress on lncRNAs, there is increasing evidence that lncRNAs are involved in the pathogenesis of endometriosis [124]. For example, the lncRNA, HOTAIR, is upregulated by estradiol binding to the estrogen receptors, ERα and ERβ. Co-regulators, including histone methyltransferases (MLL1 and MLL3) and histone acetylases in the p300–CBP family, are recruited together with estrogen receptors to bind estrogen response elements in the HOTAIR promoter in response to 17β-estradiol treatment, and they are necessary for the upregulation of HOTAIR [125]. The above interactions leading to dysregulated estrogen receptor expression in endometriosis are shown together with comodulators of estrogen signaling in Fig. 4.

#### Comodulators of Estrogen Signaling

Estrogen signaling involves recruitment of many comodulators (coactivators and corepressors) that interact with many members of the nuclear receptor-related multifunctional protein complexes, resulting in both transcriptional and epigenetic changes. The latter include chromatin density changes, histone modifications by acetylation/deacetylation and DNA methylation/demethylation. Thus, modulation of gene expression in EnSCs/MSCs depends on recruitment of comodulators crucial for the activities of the respective acetyltransferases (e.g., p300-CBP and its paralog p300; GNAT or GCN5-related N-acetyltransferase, nuclear receptor coactivator-NCOA-related histone acetyltransferase) and methyltransferases (e.g., histone-lysine N-methyl-transferases and histone-arginine N-methyltransferases) [127–129].

Interestingly, steroid receptor RNA activator (SRA) is a type of lncRNA that coordinates the functions of various transcription factors and enhances steroid receptor-dependent gene expression. As a nuclear receptor coactivator, SRA can coactivate both ERα and ERβ [130]. Low expression levels of SRA lncRNA and ERα but relatively high expression levels of SRA and ERβ have been detected in ovarian endometriotic tissues compared to normal endometrial tissues. Moreover, SRA1-small interfering RNA treatment significantly increases ERα levels but reduces ERβ levels in EnSCs. Treatment with interfering RNA also attenuates proliferation of ovarian endometriotic cells and promotes early apoptosis in these cells [131].

Sirtuins (SIRTs) possessing histone deacetylase (HDAC) activities are a good example of gene silencing by comodulators. For instance, SIRT1 represses estrogen-regulated gene expression and inhibits ligand-dependent activation of ERα [132]. Overexpression of SIRT1 may contribute to both the pathogenesis of endometriosis and P4 resistance (Fig. 4.) [133]. Interestingly, examination of eutopic end ectopic endometrial tissue obtained from the same patient has shown significantly decreased levels of SIRT1 mRNA in eutopic EnSCs compared to fEnSCs [134].

Thus, at different stages, complex and non-uniform mechanisms of estrogen/ER signaling within endometrial cells are subjected to significant modulation by epigenetic factors. Disruption of this modulation may explain the ectopic increase of EnSCs/MSCs with formation of endometriotic foci [135–138].

### P4 Signaling

**Fig. 5:**
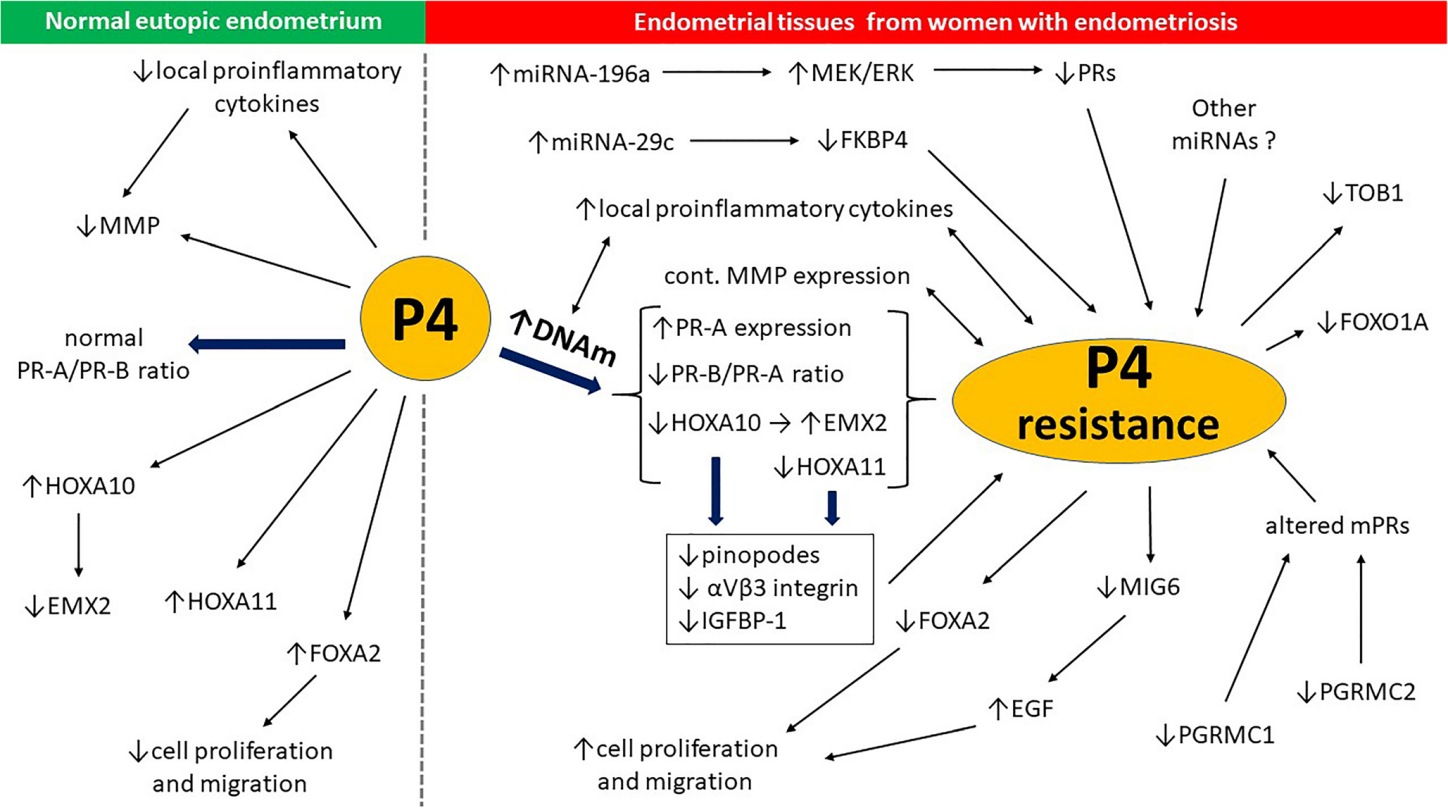
Epigenetic contributions to P4 signaling in the context of P4 resistance in endometriosis: normal eutopic endometrium vs endometrial tissues from women with endometriosis (see main text for details). *DNAm – DNA methylation; EGF – epidermal growth factor; EMX2 – homeobox protein EMX2 (Empty Spiracles Homeobox 2); FKBP4 – FK506-binding protein 4, target transcript of miRNA-29c; FOXA2, FOXO1A – transcription factors; HOXA1, HOXA11 – homeobox proteins; IGFBP-1 – insulin-like growth factor binding protein 1; MEK/ERK – the Ras/Raf/MEK/extracellular signal-regulated kinase (ERK) signaling pathway; MIG6 – mitogen inducible gene 6; MMP – matrix metalloproteinase; P4 – progesterone; PGRMC1, PGRMC2 – progesterone receptor membrane components 1 and 2, respectively; PR-A, PR-B, mPRs – progesterone receptors: A, B and membrane-bound, respectively*

Many different authors share the view that there is a pivotal role in the pathogenesis of endometriosis associated with endometrial resistance to P4 (Fig. 5.) [21, 85]. Studies of normal human endometrial tissue comprising mesenchymal stem/stromal cells and/or endometrial stromal cells (MSCs/ EnSCs) have demonstrated that prior exposure to P4 not only downregulates matrix metalloproteinase (MMP) expression but also limits the ability of locally produced proinflammatory cytokines to stimulate the expression of these enzymes. In contrast, endometrial tissues from women with endometriosis demonstrate an altered response to P4, allowing continuous expression of MMPs throughout the secretory phase [139]. Genomic activity related to the action of P4 in target tissues is normally mediated by nuclear progesterone receptor (PR). PR is expressed as two primarily functionally distinct isoforms, PR-A and PR-B, which are encoded by the same gene on chromosome 11q22-q23; however, they are transcribed from two distinct promoters [140, 141]. The isoforms PR-A and PR-B differ only in that human PR-B contains an additional 164 amino acid far N-terminal region called the “B-upstream segment” (BUS), which confers activation function 3 (AF3) activity [140]. Whereas the PR-B isoform was shown to stimulate transcriptional activity orchestrated by P4, lacking the BUS in the PR-A isoform predisposes patients to act as a dominant repressor of PR-B in many target tissues, including the endometrium [140]. Therefore, PR-B promotes uterine epithelial proliferation when not repressed by PR-A [142]. Consequently, in addition to functional abnormalities of the existing PRs, an altered PR-A/PR-B ratio might render specific target tissues responsive or resistant to P4, which could be essential for the pathogenesis and inflammatory activity of endometriosis. Both PR-B deficiency and PR-A overexpression should be considered [143]. For example, a decreased PR-B/PR-A ratio was demonstrated in endometrial cells after pretreatment with either tumor necrosis factor-alpha (TNF-α) and in peritoneal fluid obtained from women with advanced-stage endometriosis [143]. Therefore, P4 plays a crucial role in endometrial receptivity by acting through PR isoforms PR-A and PR-B. Further, in a role that may be essential for fertility, both PR isoforms regulate decidual prolactin (PRL) expression - a marker of decidualization - in differentiating human endometrial stromal cells [144]. DNA methylation and posttranscriptional silencing of target genes by miRNAs are two important epigenetic mechanisms regulating receptivity within eutopic endometrial tissue. It was demonstrated that both P4 synthesis and the expression of PR-A/PR-B in MSCs and EnSCs are epigenetically regulated by DNA methylation [145, 146]. Furthermore, studies in a primate (baboon) model for endometriosis revealed that P4 resistance in endometriosis is modulated by the altered expression of miRNA-29c and changes in the levels of its target transcript, FK506-binding protein 4 (FKBP4) [147]. Following induction of endometriosis in baboons, the mean expression of miRNA-29c in the eutopic endometrium was increased with a coexisting decrease in FKBP4 level in this tissue. Human data corroborated the baboon data and demonstrated significantly higher expression of miRNA-29c in the eutopic endometrium of women with endometriosis than what was observed in the normal endometrium of controls. Moreover, after radical laparoscopic excision of endometriosis, miRNA-29c expression in the eutopic endometrium of female patients was markedly decreased compared with the levels in preoperative eutopic tissue samples containing MSCs/ EnSCs. FKBP4 showed an inverse trend postoperatively [147]. In another study, the relationship between miRNA-196a levels and PR expression was studied in the context of the Ras/Raf/MEK/extracellular signal-regulated kinase (ERK) signaling pathway [148]. It was demonstrated that upregulation of MEK/ERK signaling by miRNA-196a is involved in epigenetic downregulation of PR in the eutopic endometrium of women with endometriosis [148]. It is very likely that other miRNAs not yet known may also be responsible for progestin resistance in endometriosis.

The combination of local (ectopic) growth and inflammation is the immanent feature of endometriosis, producing a vicious circle with further promotion of proliferation and more inflammation if not successfully treated. Considering that the prolonged stimulation by a pro-inflammatory cytokine (e.g., NF-κB) induces at least partial methylation at PR-B with concomitant PR-B downregulation in endometriotic cells, the effects of such epigenetic exclusion of local P4 receptivity may be crucial in the pathophysiology of endometriosis. A study on an immortalized endometrial stromal cell line derived from a normal woman revealed that the knockdown of PR-B by a small interfering RNA (siRNA) leads to a significant increase in proliferation [149, 150]. Being aware of the limitations of this in vitro study, the results suggested that PR-B knockdown might be responsible, at least in part, for increased proliferation and resistance to apoptosis, as seen in the eutopic and ectopic endometrium of women with endometriosis [150].

There are non-answered questions about the relationship between eutopic endometrium and ectopic endometrial foci regarding the trigger of P4-attenuated response or P4 resistance. It is still not clearly demonstrated how a defective endometrium could initiate the conditions that predispose patients to endometriosis or, alternatively, whether endometriosis produces hampered endometrial receptivity to P4 [21, 151]. It was proposed that the eutopic endometrium of ill women had an attenuated response to P4 because estrogen-responsive genes are not suppressed in their stromal cells compared to normal women in the early secretory phase of the menstrual cycle, which would suggest a phenotype of P4 resistance [152]. A potential mechanism responsible for the altered expression of the respective genes linked to decreased endometrial receptivity in endometriosis was demonstrated in an animal model. Based on mouse comparative studies, it was suggested that impaired endometrial receptivity in the endometriosis group may be caused by altered gene expression due to methylation of the homeobox protein A10 (HOXA10) and A11 (HOXA11) [151]. Hoxa10/HOXA10 and hoxa11/HOXA11 are important transcriptional moderators that either activate or repress the downstream target genes involved in uterine embryogenesis and endometrial receptivity [153]. During the normal menstrual cycle in women, the expression of both HOXA10 and HOXA11 is driven by sex steroids, peaks rapidly during the implantation window in response to rising P4 levels, and then remains elevated throughout the secretory phase [154]. This increase in HOXA10 and HOXA11 levels is not observed in patients with endometriosis [155, 156]. Considering that endogenous endometrial HOXA10 expression directly regulates endometrial expression of important factors for embryo implantation, including β3-integrin and the divergent homeobox gene emx2/EMX2, hypermethylation of hoxa10 at the peri-implantation period may predispose patients to epigenetically determined infertility [153]. Indeed, silencing hoxa10 via methylation counteracts EMX2 downregulation, and abnormally high levels of EMX2 expression were demonstrated in endometriosis. Next, the β3-integrin subunit, as a direct hoxa10 downstream target gene, was aberrantly expressed at low levels at the time of implantation in the endometrium of women with endometriosis [157, 158]. Moreover, methylation of hoxa10 and hoxa11 leads to the release of blocked expression of proinflammatory cytokines; one such group, is the interleukin-1 (IL-1) family, a group of 11 cytokines that plays a crucial role in the regulation of immune and inflammatory responses (including the conceptus-endometrium interaction) to establish pregnancy [159, 160]. It follows from the above data that altered PR expression or diminished activity identified in endometriosis as P4 resistance may be caused by aberrant epigenetic regulation of P4-responsive genes (including hoxa10 and hoxa11) in eutopic EnSCs. In turn, other mediators of endometrial receptivity that are regulated by hox genes, such as pinopodes, alphavbeta3 integrin, and IGFBP-1, are downregulated in endometriosis [154]. The relatively permanent nature of P4 target gene silencing by methylation may explain the typically observed but unsatisfactory treatments for endometriosis-related infertility [154].

According to recent studies, epigenetic modifications of another P4-regulated gene in the eutopic endometrium of endometriosis-affected individuals may be crucial in P4 resistance and disease etiology. These modifications include DNA hypermethylation with altered gene expression relevant to endometrial function/dysfunction, including cell migration and proliferation [161]. Functional loss of the respective chromosomal regions due to hypermethylation resulting in downregulation of proteins that regulate the cell cycle in the endometrial tissue of women with endometriosis, such as transcription factors FOXO1A and FOXA2, protein ErbB-2 (TOB1) and mitogen inducible gene 6 (MIG6), has been well documented [98, 162, 163]. The latter is a negative regulator of epidermal growth factor (EGF), and it promotes proliferation and migration of EnSCs [164, 165]. Similarly, FOXA2 deficiency was found to contribute to cell proliferation and migration in eutopic endometrium from patients with endometriosis. Thus, these women may participate in the “metastatic” process of eutopic endometrium transitioning into ectopic loci. Unlike in endometriosis, FOXA2 expression can be induced by P4 in a healthy endometrium [166].

The discovery of membrane-bound progesterone receptors (mPRs: mPRα–ϵ) of the progestin and adipoQ receptor (PAQR) family, including progesterone receptor membrane component 1 and 2 (PGRMC1 and PGRMC2), suggests that 7-transmembrane receptors coupled to G proteins may be involved in fast cell surface-initiated actions by P4 that – unlike the actions of P4 via classical PRs – occur over a time scale of minutes [167–169]. It was demonstrated that changes in the balance between PGRMC1 and PGRMC2 may participate in and/or regulate EnSCs survival (mitosis and apoptosis) throughout the menstrual cycle [170].

There is increasing evidence that PGRMC2 facilitates progestational effects within the endometrial glands. Thus, abnormal expression and/or intracellular localization of PGRMC2 may contribute to the blunted response to P4, leading to abnormal endometrial function and decreased fertility observed in subjects with endometriosis. Indeed, reduced levels and abnormal intracellular localization patterns of PGRMC2 in the endometrium of primates (macaques) afflicted with advanced endometriosis were also observed [171]. This may suggest that endometrial alterations in membrane-bound PGRMC2 may contribute to the phenomenon of P4 resistance. Similarly, altered expression of mPRs in MSCs and EnSCs may be linked to infertility and endometriosis. The expression of the progesterone membrane receptor (mPR-β) in the endometrial tissue of patients with recurrent spontaneous abortions was significantly lower in comparison with that of the normal control group, whereas endometrial PGRMC-1 and PGRMC-2 expression was reported to be downregulated in secretory phase endometrium from women with advanced stage endometriosis [172, 173].

Increasing evidence suggests that intracellular signaling pathways related to mPR involve rapid nongenomic actions that may be epigenetically controlled throughout the menstrual cycle [174, 175]. It has been proven that a mitogen-activated protein kinase (MAPK), a key enzyme in mPR signal transduction pathway (the Ras-Raf-MEK-ERK pathway) affects chromatin modifications in multiple ways producing histone modifications through phosphorylation of transcription factors including the cAMP-response-element binding factor (CREB) and NF-κB, which recruit chromatin-modifying complexes and through direct phosphorylation of histones [176–178]. However, further in vivo experiments with specific mPR agonists and antagonists are needed to elucidate the strength of these epigenetic influences in the context of both the pathogenesis of endometriosis and new treatment methods. The mentioned epigenetic contributions to P4 signaling in the context of endometriosis are summarized in Fig. 5.

## Concluding Remarks

Knowledge about epigenetic mechanisms has significantly increased in recent years. This also applies to endometriosis, in which general endocrine mechanisms have been described several decades ago but still without clarification of the triggering factors [179]. There is currently no doubt that modifications of MSCs and/or EnSCs that comprise histone variants, posttranslational modifications of amino acids on the amino-terminal tail histones and covalent modifications of DNA bases are involved in the etiopathogenesis of endometriosis or at least significantly affect the course of endometriosis [78, 138, 180, 181]. Epigenetic alterations of the transcription factors of estrogen and P4 signaling pathways in MSCs are robust in endometriotic tissue [182]. Moreover, these processes leading to P4 resistance and ER subtypes imbalances are not limited to nuclear PRs and ERs but also include membrane-bound PR and G protein-coupled ER 1 [176, 183]. Current therapies for endometriosis cannot completely cure the disease, and patients present with high recurrence rates. Therefore, novel medical approaches are needed. Perspectives for the future may include MSCs and EnSCs as the potential targets of epigenetic therapies in the prevention and treatment of endometriosis [78, 184]. Potential advantages of single cell molecular profiling in endometrium and in endometriotic foci should be considered [184]. Moreover, in cases of higher invasiveness of MSCs and/or EnSCs in ectopic locations, manifested in higher proliferation, migration and angiogenic ability in comparison with eutopic MSCs/EnSCs, a tyrosine kinase inhibitors may be promising. These compounds are able to revert the increased proliferative, migratory and angiogenic phenotype of ectopic endometrial MSCs [185]. Considering that MSCs-related angiogenesis play important role in the survival and proliferation of both eutopic and ectopic endometrial tissue, inhibition of the formation of new blood vessels represents a rationale for targeted antiangiogenic approach in the treatment of endometriosis [186].

In addition, as only differentiated endometrial cells, and not endometrial MSCs, possess fully expressed steroid hormone receptors, the effectiveness of hypoestrogenism-inducing therapies may depend on the number of EnSCs in menstrual blood and/or cyclic mobilization of bone marrow derived stem cells (MSCs) [186]. Epigenetic modifications of gene expression regulation by dissecting the respective interactions between environmental factors and histone modification, DNA methylation or miRNA and lncRNA expressions influencing estrogen and P4 signaling may be crucial in developing new remedies for endometriosis [187]. Thus, therapeutic modulation of epigenetic drivers (epigenetic effectors) of endometriosis should be considered. However, ensuring the safety of such treatment for the patient remains a task for the future [187].

## Data Availability

Not applicable.
